# Vilsmeier–Haack complex formation by Fe_3_O_4_@SiO_2_@CS@POCl_2-x_/DMF: an efficient catalyst for conversion of epoxides to β-bromoformates

**DOI:** 10.55730/1300-0527.3717

**Published:** 2024-10-14

**Authors:** Farzaneh EBRAHIMZADEH

**Affiliations:** Department of Chemistry, Marvdasht Branch, Islamic Azad University, Marvdasht, Iran

**Keywords:** Magnetic nanocatalyst, Vilsmeier-Haack complex, β-bromoformates epoxide, bromide, core-shell structure

## Abstract

The in situ generation of the Vilsmeier–Haack complex was studied by combining Fe_3_O_4_@SiO_2_@CS@POCl_2-x_ with *N*,*N-*dimethylformamide (DMF), demonstrating remarkable efficiency and regioselectivity in transforming epoxides into β-bromoformates. The reported yields were notably high and were achieved under mild reaction conditions. Interestingly, the removal of DMF led to the synthesis of *vic*-dihalide compounds. The product of this transformation depended on the specific details of how the experiment was done, highlighting how sensitive the process is to different ways of conducting the experiment. The use of a magnetic core-shell catalyst, Fe_3_O_4_@SiO_2_@CS@POCl_2-x_, facilitates a straightforward work-up procedure, simplifying the isolation of the desired products. Furthermore, the reactions were conducted under clean and neutral conditions, contributing to their environmentally friendly nature.

## 1. Introduction

The Vilsmeier–Haack reagent [1,2] was made by mixing phosphoryl chloride (POCl_3_) with certain formamides such as *N,N*-dimethylformamide (DMF). This method, initially designed for formylating aromatic compounds, has since progressed into a versatile tool due to its broad substrate scope and mild reaction conditions [3]. In the Vilsmeier–Haack reaction, the proposed process involves breaking the P-Cl bond, leading to unique structures, especially an ionic one [4,5]. Moreover, formamide compounds like *N*-methyl formamide [6], *N*-formyl piperidine [7], and *N*-formyl indoline [8], along with other acid chlorides such as COCl_2_ [9,10], CHCl_3_ [10], PCl_5_ [11], SOCl_2_ [12], CH_3_COCl [13], ArCOCl [14], ArSO_2_Cl [15], and POCl_3_/DMF [16], have been used in Vilsmeier–Haack reactions. This broadens the range of chemicals capable of forming the Vilsmeier–Haack complex [17]. This flexibility allows for different reaction conditions and compatibility with various substances.

The chemical synthesis of *vic*-dihalides [18] and β-haloformates [19] is crucial, offering versatile strategies that produce essential intermediates. Various methods have been explored for their synthesis, including the ring-opening of epoxides [20,21], the transformation of alcohols and alkyl halides [22], carboxylic acid [22,23], or ester modification utilizing a range of catalysts such as iridium complex [8], magnesium oxide [24], silphos [25], phosphorous-based ionic liquids [22], and *N-*halo compound [18,21]. These versatile synthetic strategies not only enable the production of vital intermediates but also show potential for advancing medicinal chemistry and contributing to healthcare innovations [26]. Despite the existence of various formylation reagents, their application is nuanced, each presenting distinct advantages and limitations. The experimental conditions, often demanding in terms of medium acidity, formylation temperatures, and potential side reactions, require careful optimization, especially for substrates with specific sensitivities or multifunctionality [27].

Among the abovementioned substrates, epoxides stand out as highly valuable and versatile intermediates in both natural processes and modern organic synthesis. Epoxides, with their accessible and functionalized three-membered heterocycles, exhibit high reactivity due to ring strain and activation by Lewis acid catalysts. This strategic approach allows for their flexible reactivity, making them essential in synthesizing complex organic molecules for decades. The inherent ring strain and electrophilicity of epoxides also make them highly attractive as building blocks in fundamental organic reactions [28]. Despite these advantages, achieving precise control over the regioselectivity of disubstituted epoxide transformations poses a notable challenge. The intricate balance of factors makes epoxides not only readily available and functionalizable but also presents a nuanced landscape for researchers, emphasizing their crucial role in advancing the field of organic chemistry [29].

**Figure 1:**
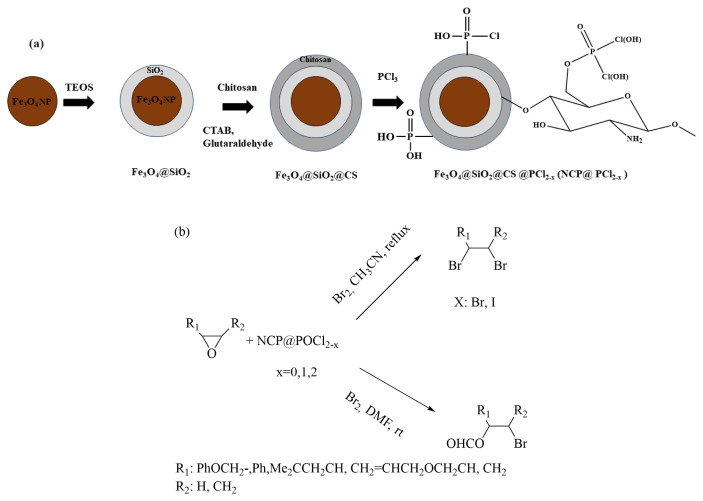
Schematic representation of (a) steps for the synthesis of NCP@POCl_2-x_ and (b) the epoxide reaction with NCP@POCl_2-x_ in the presence or absence of DMF.

In this study, we explore the capability of in situ formation of the Vilsmeier–Haack complex using a core-shell nanomagnetic catalyst, Fe_3_O_4_@SiO_2_@CS@POCl_2-x_ [30–34], referred to as NCP@POCl_2-x_/DMF, to facilitate a one-step conversion. We study the method’s applicability and limitations, including the optimization of the solvent, operation temperature, and amount of catalyst. The outcomes of this optimization reveal the successful formation of β-bromoformates and the *vic*-dibromide compound, as illustrated in Figure 1. The versatility demonstrated in the one-step conversion process emphasizes the significance of the Vilsmeier–Haack complex. Importantly, this research adds to the ongoing efforts to discover efficient synthetic methodologies, showcasing potential applications across various domains in organic chemistry.

## 2. Materials and methods

### 2.1. Materials and instruments

All chemicals utilized in this study were obtained from Merck (Darmstadt, Germany) or Fluka (Buchs, Switzerland). The progression of the reactions was monitored using thin-layer chromatography (TLC) with silica gel SIL G-UV 254 plates (Merck, Darmstadt, Germany). A GC model 7890 device coupled with the 5977A MSD system (Agilent Technologies, Santa Clara, CA, USA) was employed for analyses. Column chromatography was conducted with silica gel 60. Product identities were established through a comparison of their physical and spectral data with literature references. Fourier transform infrared (FTIR) spectra were obtained using a DR-8001 spectrometer (Shimadzu, Tokyo, Japan), while nuclear magnetic resonance (NMR) spectra were recorded on an Avance DPX 250MHz instrument (Bruker, Billerica, MA, USA). Additionally, mass spectrometry (MS) analyses were performed using the 7890B gas chromatography system coupled with the 5977A MSD system (Agilent Technologies). These analytical techniques contributed to the accurate characterization of the synthesized compounds.

### 2.2. General procedure for synthesis of β-bromoformates or *vic*-dibromo compound

Oxirane (1 mmol) was dissolved in 10 mL of acetonitrile (CH_3_CN) or DMF and heated to 80 °C under an argon atmosphere. Subsequently, 0.9 g of the NCP@POCl_2-x_ catalyst was gradually added to the mixture and allowed to mix for 3 h. The mixture was then cooled to below 40 °C and the slow addition of 2 mmol (0.4 g) of bromine (Br_2_) was carried out. The reaction mixture was stirred for the specified duration under reflux conditions as optimized in Table 1 and further described in Table 2. The progress of the reaction was monitored using TLC analysis. In the case of the DMF solvent, the entire reaction could also continue at room temperature. After the completion of the reaction, the NCP@POCl_2-x_ catalyst was separated using an external magnetic force. The residual reaction mixture was diluted with dichloromethane (30 mL) and subjected to sequential washes with water (3 × 25 mL), brine (1 × 25 mL), and additional water (1 × 25 mL). The organic layer was then dried using anhydrous Na_2_SO_4_, filtered, and concentrated under vacuum. The resulting products were further purified via column chromatography using a 10% ethyl acetate-petroleum ether mixture. The spectra for all products are provided in the Supporting Information, specifically in terms of the procedure for converting epoxide to β-bromoformates or *vic*-dibromo compounds.

## 3. Results and discussion

**Figure 2:**
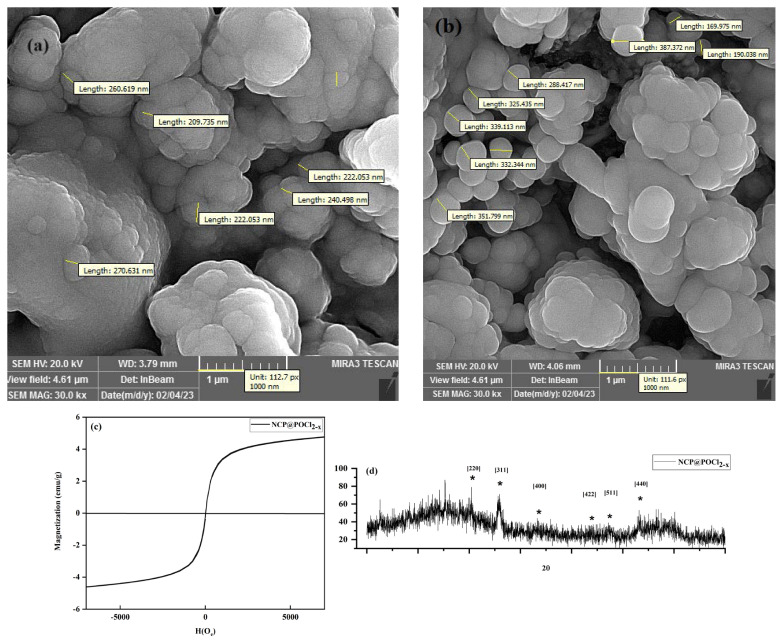
Field emission scanning electron microscopy images of (a) NCP and (b) NCP@PCl_2-x_ together with (c) vibrating sample magnetometry analysis and (d) X-ray diffraction data [30].

The comparison of field emission scanning electron microscopy images between NCP and NCP@POCl_2-x_ is illustrated in Figures 2a and 2b. The images reveal a noticeable increase in nanoparticle size upon the incorporation of POCl_2-x_ groups in the surface’s last layer. This suggests that chitosan is effectively connected to the magnetic SiO_2_, indicating a structural modification in the nanoparticles. The changes in nanoparticle size provide insights into the successful integration of the POCl_2-x_ layer and the resulting alterations in the surface characteristics of the magnetic nanocomposite. The vibrating sample magnetometry analysis of the NCP/POCl_2-x_, shown in Figure 2c, confirmed the material’s strong magnetic behavior, indicating its suitability for applications requiring efficient separation under magnetic fields. Following this, the X-ray diffraction data depicted in Figure 2d revealed distinct peaks corresponding to Fe_3_O_4_ nanoparticles (JCPDS Card No. 19-0629) at 30.08°, 35.74°, 43.4°, 54.09°, and 63.46°, which correlate to the [220], [311], [400], [422], [511], and [440] crystal planes, respectively. The reduced intensity of these peaks is attributed to the core-shell structure and the presence of an organic layer around the Fe_3_O_4_ nanoparticles, likely contributing to the nanocomposite’s stability and enhanced performance. These data were fully reported in our previous work [30].

**Figure 3:**
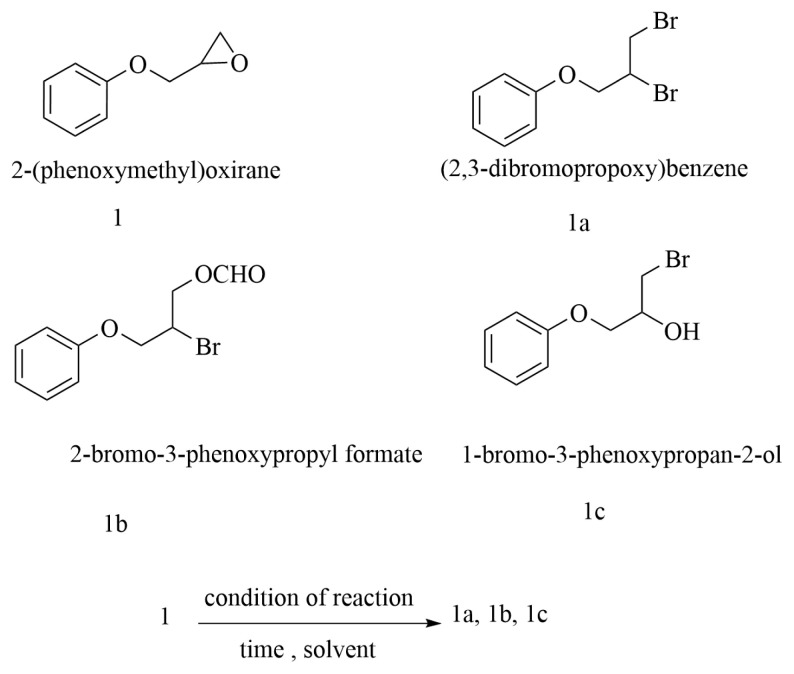
Schematic representation of phenyl glycidyl ether: optimization conditions and proposed products.

For a better understanding, an extended investigation was conducted to explore the influence of both solvent choice and the molar ratio of the magnetic catalyst on the ring-opening reaction of the epoxides. To illustrate, a reaction involving phenyl glycidyl ether (2-(phenoxymethyl)oxirane) (1 mmol) was conducted using a mixture of NCP@POCl_2-x_ (0.5 mmol) and Br_2_ (2 mmol) under various conditions, as depicted in Figure 3. The details of these conditions are presented in Table 1. The optimal molar ratio of NCP@POCl_2-x_ to the substrate was determined to be 0.5:1 mmol. Various solvents were used to systematically investigate their effects on the reaction outcomes. In cases where the solvent contained residual moisture, a mixture of products, including both β-halo alcohol and *vic*-dibromide, was observed. Conversely, the use of dry and pure solvent resulted in the synthesis of a singular product. Remarkably, the use of DMF led to the formation of a distinct product compared to other solvents. This suggests that the nature of the solvent plays a significant role in influencing the reaction mechanism and the formation of intermediates. The experimental findings highlighted the efficacy of dry polar solvents, particularly acetonitrile, due to their enhanced performance with ionic intermediates. Remarkably, under reflux conditions in dry acetonitrile, the reaction exhibited complete conversion within 5 h, yielding (2,3-dibromopropoxy)benzene with 100% conversion (Table 1, entry 9). It is noteworthy that, in the presence of DMF, a different product was obtained, achieving 100% conversion and 90% isolated yield of the β-bromoformate product, as confirmed by TLC, GC, and ^1^H NMR analysis. The identified product was established as 2-bromo-3-phenoxy propyl formate. Results indicated that the reaction is temperature-independent, as it demonstrated comparable outcomes at both room temperature and 80 °C (Table 1, entries 10 and 11). The observed variations in product formation emphasize the substantial influence of both solvent composition and reaction conditions on the outcomes of the ring-opening reaction of epoxides with bromine in the presence of NCP@POCl_2-x_.

The study extended to a diverse range of aliphatic and cyclic epoxides, with a specific focus on those incorporating electron-withdrawing substituents. These varied epoxides underwent reactions in either CH_3_CN (dry) or DMF. The intentional inclusion of electron-withdrawing substituents aimed to assess the catalytic system’s performance under more challenging conditions, thereby highlighting its versatility and potential for synthesizing a wide array of compounds. In the presence of dry CH_3_CN, as mentioned earlier, only the *vic*-dibromo compound was synthesized. Conversely, with DMF as the solvent, the product changed exclusively to β-bromoformate. The results for different epoxides with diverse structures are presented in Table 2.

Detailed results of ring-opening reactions with various substituted epoxides and NCP@POCl_2-x_, presented in Table 2, reveal the impact of different substituents on regioselectivity, underscoring the versatility and scope of this synthetic approach. The consistent and high yields underscore the efficiency of the method in selectively generating products with bromination preferentially occurring at more hindered sites across diverse epoxide substrates.

**Figure 4:**
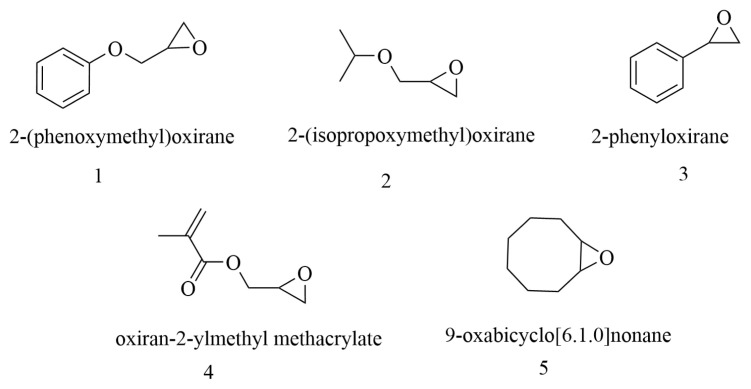
Substrate employed in reaction as detailed in Table 2.

All substrates used in the reaction are shown in Figure 4 and are numbered as represented in Table 2.

**Figure 5:**
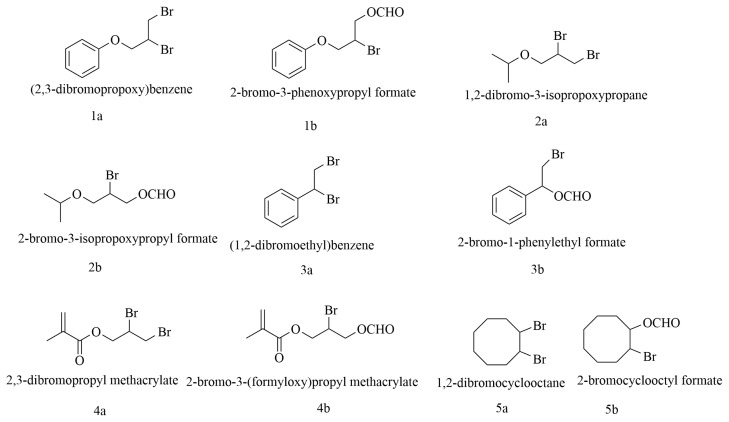
Chemical structure of reaction products as illustrated in Table 2.

The reaction products, as represented in Figure 5 and listed in Table 2, were accurately characterized using various spectroscopic techniques, including boiling point (bp), melting point (mp), ^13^C NMR, and ^1^H NMR analyses. This extensive dataset was subsequently compared with findings previously reported in the literature, with more details on the products available in the Supporting Information section.

**Figure 6:**
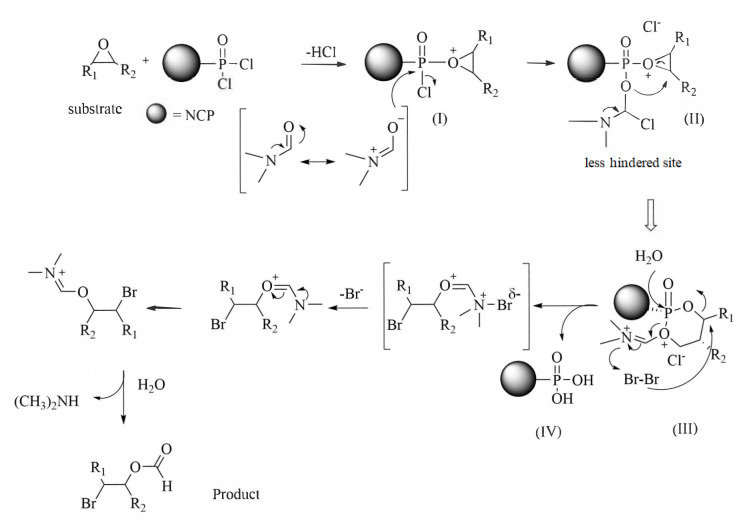
Proposed mechanism for the ring-opening reaction of epoxide/Br_2_ in the presence of NCP@POCl_2-x_/DMF.

Functional groups, including simple aromatic rings, ether, esters, and alkene groups (see Table 2, entries 1, 2, and 5), demonstrate stability under the reaction conditions. The regioselectivity observed is likely influenced by a combination of steric and electronic factors. The pronounced regioselectivity observed in the formation of β-bromoformate can be attributed to the bromine ion preferentially attacking the less hindered carbon of the epoxide. This phenomenon is associated with the reaction mechanism, and the formation of the Vilsmeier–Haack complex, as illustrated in Figure 6, is proposed as the mechanism for the reaction. As shown, the process initiates with the epoxide attacking the NCP@POCl_2-x_. The oxygen in the epoxide group shows a preference for attachment to the phosphate group, leading to the formation of an oxonium intermediate (I). Simultaneously, DMF in the surrounding environment undergoes rearrangement and binds to phosphorus, generating intermediate (II). This Vilsmeier–Haack complex, represented by intermediate (II), prompts a rearrangement that forms a six-membered ring, with two oxygen atoms positioned adjacent to the phosphorus group. During this rearrangement, an attack is facilitated by the oxygen associated with DMF on the oxirane ring at a less hindered site, resulting in the formation of intermediate (III). In the presence of Br_2_, intermediate (III) becomes activated, leading to the separation of NCP@PO(OH)_2_ (IV). This separated entity continues the cycle of the reaction, allowing for its repetition. According to this mechanism, the high regioselectivity in the ring-opening reactions of epoxides is explained, resulting in the exclusive formation of a single isomer. This emphasizes the precision and reliability of the reaction methodology.

**Figure 7:**
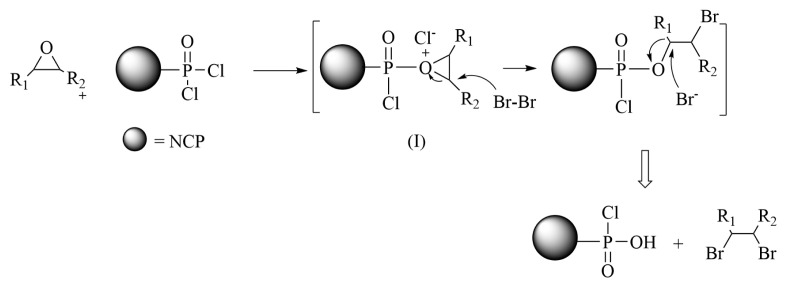
Proposed mechanism for the ring-opening reaction of epoxide/Br_2_ in the presence of NCP@POCl_2-x_/CH_3_CN.

In the absence of DMF, the coexistence of epoxide, NCP@POCl_2-x_, and bromine resulted in the formation of a *vic*-dihalo compound. The proposed mechanism, illustrated in Figure 7, closely resembles the previously described process. However, in the absence of DMF, bromine attacks intermediate (I) and the reaction proceeds through the nucleophilic attack of bromide ions in the solution. In the presence of water, H_2_O also acts as a nucleophile, leading to the observation of a mixed product. The absence of DMF significantly influences the reaction outcome, resulting in the formation of a different predicted product. This emphasizes the pivotal role of DMF in the reaction and underscores its impact on the observed products.

The recovery efficiency of NCP/POCl_2-x_ was evaluated over four cycles to ensure consistent reaction yields. The results indicated that while the presence of chlorine atoms in NCP@POCl_2-x_ is necessary, the reaction did not perform well with only hydroxyl groups on the surface of the nanocomposite. To recover NCP/PO(OH)_2_ and convert it back to NCP@POCl_2_, the process begins with a magnetic filtration method to separate NCP/PO(OH)_2_ from the reaction mixture. After filtration, the recovered material is thoroughly washed with dichloromethane and ethanol for further purification. The washed NCP/PO(OH)_2_ is then dried at a controlled temperature of 60–70 °C to remove any remaining solvents and moisture. Finally, to convert NCP/PO(OH)_2_ back to NCP@POCl_2-x_, it was treated with phosphorus oxychloride (POCl_3_). This recovery procedure was repeated four times to evaluate the effectiveness of the NCP/POCl_2-x_ recovery method, as detailed in Table 3.

## 4. Conclusion

The use of NCP@POCl_2-x_ as a catalyst in the ring-opening reaction of epoxides has demonstrated remarkable versatility, yielding a variety of products depending on the solvent used. Notably, with DMF as the solvent, the catalyst facilitates the formation of the Vilsmeier–Haack complex, thereby expanding the scope of potential reactions. The practical advantages of NCP@POCl_2-x_ include operational simplicity, easy separation of the catalyst from the reaction mixture using external magnetic force, enhanced product yields, accelerated reaction rates, and impressive regioselectivity. Ultimately, this study highlights the promising potential of NCP@POCl_2-x_ as a versatile catalyst in various solvent environments, opening new avenues for its application in tailored synthetic methodologies.

## Supplementary Information



## Figures and Tables

**Table 1:** Optimization of reaction conditions for the ring-opening reaction of phenyl glycidyl ether using Br_2_ and NCP@POCl_2-x_.

Entry	Solvent	Time (h)	Condition of reaction	Product (%)	GC yield (%)
1	THF	24	NCP@POCl_2-x_ (2g), Br_2_ (2 mmol), reflux	1a&1c	25% mixed product
2	CHCl_3_	24	NCP@POCl_2-x_ (2g), Br_2_ (2 mmol), rt	1a&1c	15% mixed product
3	CH_2_Cl_2_	24	NCP@POCl_2-x_ (2g), Br_2_(2 mmol), rt	1a&1c	Less than 10% mixed product
4	EtOAc	24	NCP@POCl_2-x_ (2g), Br_2_(2 mmol), rt	1a&1c	31% mixed product
5	CH_3_CN	24	NCP@POCl_2-x_ (2g), Br_2_(2mmol), rt	1a&1c	36% mixed product
6	EtOAc	24	NCP@POCl_2-x_ (2g), Br_2_(2 mmol), reflux	1a&1c	73% mixed product
7	CH_3_CN	24	NCP@POCl_2-x_ (2g), Br_2_(2mmol), reflux	1a&1c	100% mixed product
8	CH_3_CN (dry)	5	NCP@POCl_2-x_ (2g), Br_2_(2mmol), reflux	1a	100%
9	CH_3_CN (dry)	5	NCP@POCl_2-x_ (1g), Br_2_(2mmol), reflux	1a	100%
10	DMF	24	NCP@POCl_2-x_ (1g), Br_2_(2mmol), rt	1b	100%
11	DMF	10	NCP@POCl_2-x_ (1g), Br_2_ (2mmol), reflux	1b	100%

**Table 2:** Reaction of epoxides/Br_2_ in the presence of NCP@POCl_2-x_ in DMF or CH_3_CN.

Entry	Substrate	Solvent	Product	Time (h)	Yield (%)
1	1	CH_3_CN(dry)	1a	5	92
		DMF	1b	10	91
2	2	CH_3_CN(dry)	2a	5	96
		DMF	2b	24	89
3	1	CH_3_CN(dry)	1a	3	94
		DMF	1b	24	90
5	4	CH_3_CN(dry)	4a	3	94
		DMF	4b	24	92
6	5	CH_3_CN(dry)	5a	24	90
		DMF	5b	24	86

**Table 3:** Evaluation of catalyst recovery process in the formylation of compound **1** in the presence of DMF/Br_2_.

Entry	Runs	Yield (%)
1	first	90
2	second	89
3	third	90
4	forth	83
