# Supplementary material for: Vilsmeier–Haack complex formation by Fe3O4@SiO2@CS@POCl2-x/DMF: an efficient catalyst for conversion of epoxides to β-bromoformates
Source: Turk J Chem. 2024 Oct 14;49(2):133–42. doi: 10.55730/1300-0527.3717 (PMC12068672; doi:10.55730/1300-0527.3717)
Supplement: Supplementary file 1 [file supp-tjc-49-02-133.docx]

Supporting Information

Vilsmeier-Haack complex formation by Fe_3_O_4_@SiO_2_@CS@POCl_2-x_ /DMF: Efficient catalyst for conversion of epoxide to β-bromoformates

Farzaneh EBRAHIMZADEH ^1, *^

^1^ Department of Chemistry, Marvdasht Branch, Islamic Azad University, Marvdasht, Iran,

*Correspondence: polychemfar@miau.ac.ir

ORCIDs:

First AUTHOR: https://orcid.org/0000-0001-5825-7288

Contents

[The procedures and spectral data 1](#_Toc156636728)

[Method of synthesis of NCP and NCP@POCl_2-x_ 1](#_Toc156636729)

[Representative procedure for the synthesis of (2,3-Dibromopropoxy)benzene (1a) 1](#_Toc156636730)

[Representative procedure for the synthesis of 2-Bromo-3-phenoxypropyl formate (1b) 2](#_Toc156636731)

[2.2.1. (2,3-Dibromopropoxy)benzene (1a)^[3]^: 2](#_Toc156636732)

[2.2.2. 2-Bromo-3-phenoxypropyl formate (1b): ^[4]^ 4](#_Toc156636733)

[2.2.3. 1,2-Dibromo-3-isopropoxypropane (2a)^[3],[5]^ 5](#_Toc156636734)

[2.2.4. 2-Bromo-3-isopropoxypropyl formate (2b) ^[4]^ 5](#_Toc156636735)

[2.2.5. (1,2-Dibromoethyl) benzene (3a)^[3, 6]^ 6](#_Toc156636736)

[2.2.6. 2-Bromo-1-phenylethyl formate (3b) ^[4]^ 8](#_Toc156636737)

[2.2.7. 2,3-Dibromopropyl methacrylate (4a)^[3, 7, 8]^ 10](#_Toc156636738)

[2.2.8. 2-Bromo-3-(formyloxy)propyl methacrylate(4b)^[4]^ 11](#_Toc156636739)

[2.2.9. 2-bromocyclooctyl formate (5b)^[3]^ 13](#_Toc156636740)

[References 15](#_Toc156636741)

# **The procedures and spectral data**

# **Method of synthesis of NCP and NCP@POCl_2-x_**

The purification of chitosan prior to nanocomposite synthesis and the synthesis procedure for Fe_3_O_4_@SiO_2_@CS (NCP) and NCP@POCl_2-x_ was recently documented in the literature,^1, 2^ outlining the specific steps involved in NCP preparation**.**

## **Representative procedure for the synthesis of (2,3-Dibromopropoxy) benzene (1a)**

2-(phenoxymethyl)oxirane (1 mmol, 0.15 g) was dissolved in 10 ml of acetonitrile (CH_3_CN) and heating at 80˚C under an argon atmosphere. 0.9 g of the NCP@POCl_2-x_ catalyst was gradually added to the mixture and allowed to mix for 3h. Subsequently, the mixture was cooled to below 40˚C, and a slow addition of 2 mmol (0.4 g) of bromine (Br_2_) was carried out. The reaction mixture was stirred for 5h. The progress of the reaction was monitored using thin-layer chromatographic analysis (TLC). After the completion of the reaction, the NCP@POCl_2-x_ catalyst was separated using an external magnetic force. The residual reaction mixture was diluted with dichloromethane (30 ml) and subjected to sequential washes with water (3 × 25 ml), brine (1 × 25 ml), and additional water (1 × 25 ml). The organic layer was then dried using anhydrous Na_2_SO_4_, filtered, and concentrated under vacuum. The resulting products were further purified via column chromatography using a 10% ethyl acetate-petroleum ether mixture.the conversion of yellow crystal product is 100% and and isolated yield of product was earnd 92% ^3^.

## **Representative procedure for the synthesis of 2-Bromo-3-phenoxypropyl formate (1b)**

2-(phenoxymethyl)oxirane (1 mmol, 0.15g) was dissolved in 10 ml of *N,N*-dimethylformamide (DMF) and heating at 80˚C under an argon atmosphere. 0.9 g of the NCP@POCl_2-x_ catalyst was gradually added to the mixture and allowed to mix for 3h. Subsequently, the mixture was cooled to below 40˚C, and a slow addition of 2 mmol (0.4 g) of bromine (Br_2_) was carried out. The reaction mixture was stirred for 10h. The progress of the reaction was monitored using thin-layer chromatographic (TLC) analysis. the reaction also could continue at room temperature. After the completion of the reaction, the NCP@POCl_2-x_ catalyst was separated using an external magnetic force. The residual reaction mixture was diluted with dichloromethane (30 ml) and subjected to sequential washes with water (3 × 25 ml), brine (1 × 25 ml), and additional water (1 × 25 ml). The organic layer was then dried using anhydrous Na_2_SO_4_, filtered, and concentrated under vacuum. The resulting products were further purified via column chromatography using a 10% ethyl acetate-petroleum ether mixture. ^4^

Here, the spectra for all products are shown in the material and method section, specifically in the procedure for converting epoxide to β-Bromoformates or *vic*-dibromo compound.

**2.2.1. (2,3-Dibromopropoxy)benzene (1a)**^3^**:**

Yellow crystal, Isolated yield: 92%, m.p: 77-79°C, ^1^HNMR (in CDCl_3,_ 250 MHz): Chemical shifts (δ ppm): 7.64-7.10 (m, J =12.455, J =12.162 Hz, 5H, aromatic), 5.88-5.82 (m, 1H), 3.72 (d, J=8.533 Hz ,2H), 3.48 (d, J=8.522 Hz, 2H). ^13^CNMR Chemical shifts (δ ppm): 160.17 (1C, CHO) , 130.42-129.61 (2C),120.80 (1C), 114.40 (2C), 79.78(1C), 47.79 (1C, C-Br), 32.46 (1C). Anal. Calcd for C_9_H_10_Br_2_O (MW= 293.97g/mol): C, 36.76%; H, 3.43%,; Found: C, 38.75%; H, 3.23%,. MS(m/z)(g/mol): 293.9251 (100%, C_9_H_10_Br_2_O), 291.9364 (49.8%) , 234.894 (51.2%, C_6_H_3_Br_2_), 214.1332 (12.7%, C_9_H_10_BrO), 134.2828 (8.1%, C_9_H_10_O), 78.1311 (5.2%, C_6_H_6_)


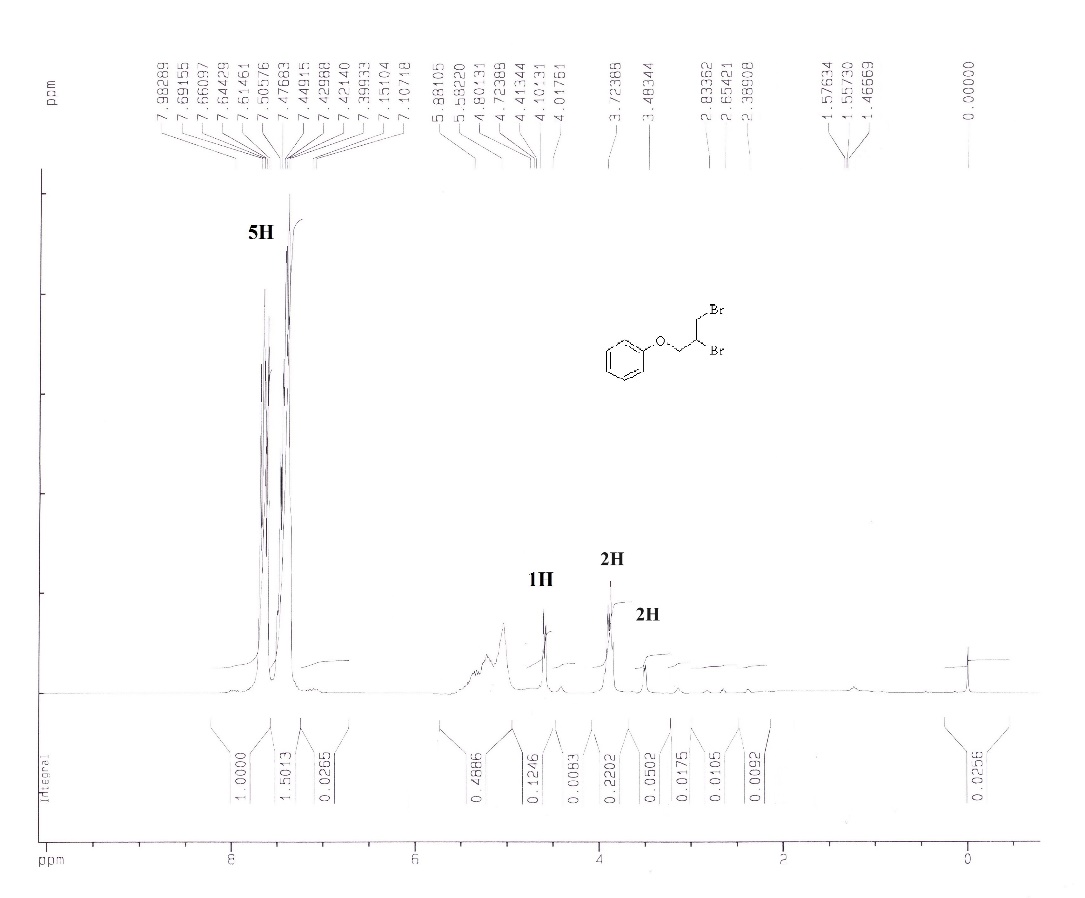


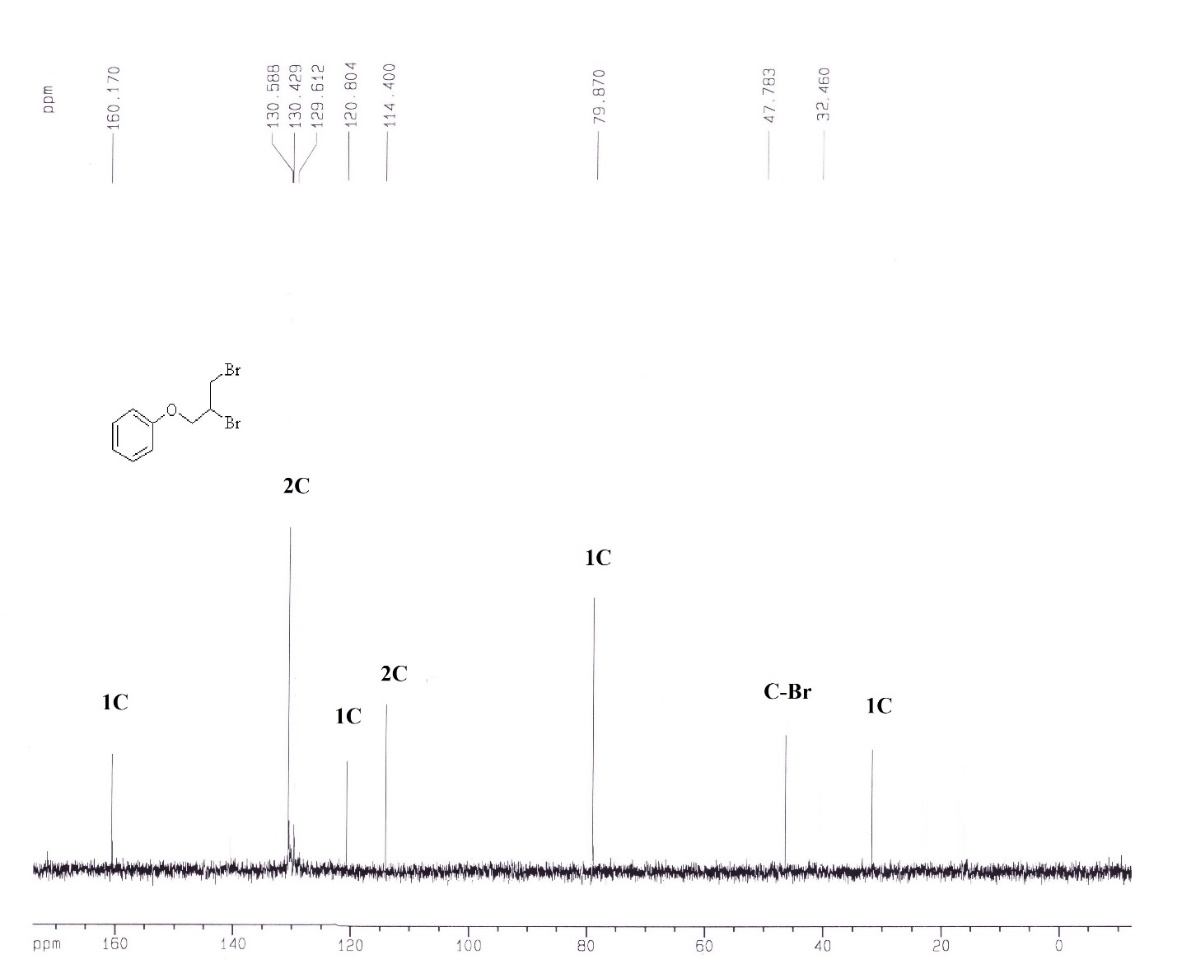


**2.2.2. 2-Bromo-3-phenoxypropyl formate (1b):** ^4^

Light yellow crystal ,isolated yield: 91%, m.p: 91-95°C, FTIR (υ/cm^-1^): 3431, 3078(=C-H), , 2931(C-H), 2869, 1714(C=O), 1598(C=C), 1490, 1239, 1145(C-O), 753 cm^-1^. ^1^HNMR (in CDCl_3_, 250 MHz): Chemical shifts (δ ppm): 8.19 (s, 1H, CHO), 7.46-7.19 (m, J =7.712 Hz, J =6.630 Hz, 5H, aromatic), 5.52(m, 1H), 4.79-4.70 (m, J=7.392 Hz, 2H), 4.21 (d, J=4.068 Hz, 1H).^13^CNMR Chemical shifts (δ ppm): 163.86 (1C), 163.67(1C), 131.51-130.66 (3C), 114.64-114.00 (2C), 78.09-77.59 (2C), 69.51 (1C), 40.132 (1C, C-Br). Anal. Calcd for C_10_H_11_BrO_2_ (MW=259.10 g/mol): C, 46.36%,; H, 4.28%. Found: C, 48.19%; H, 4.32%. MS(m/z)(g/mol): 257.9901 (100%, C_10_H_11_BrO_2_), 198.1564 (7.2%, C_9_H_11_BrO), 181.0255 (32.16%, C_9_H_11_Br), 165.1933 (65.7%, C_10_H_11_O_2_), 149.9426 (43.5%, C_9_H_9_O), 45.0211 (2.1%, CHO_2_).


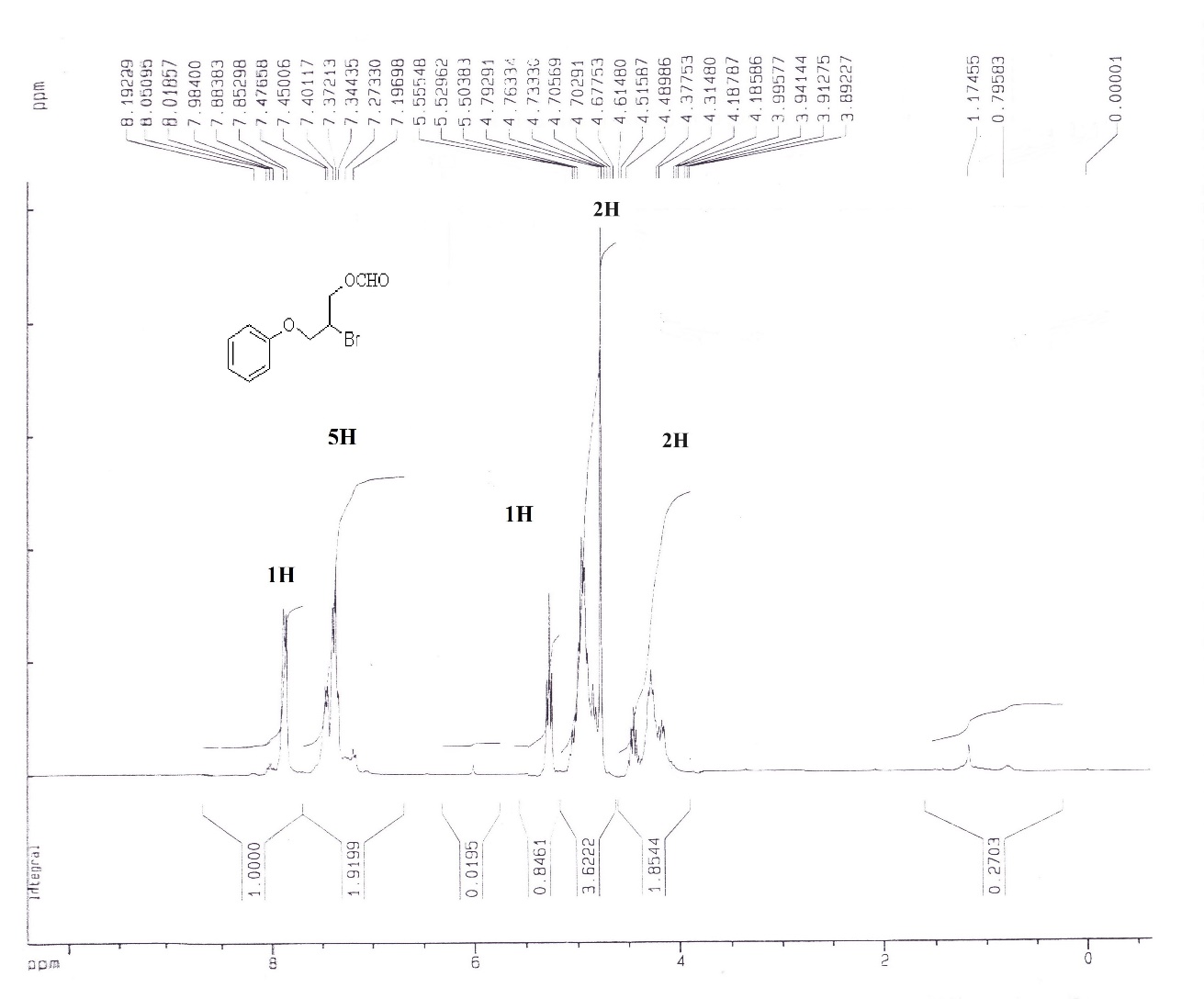


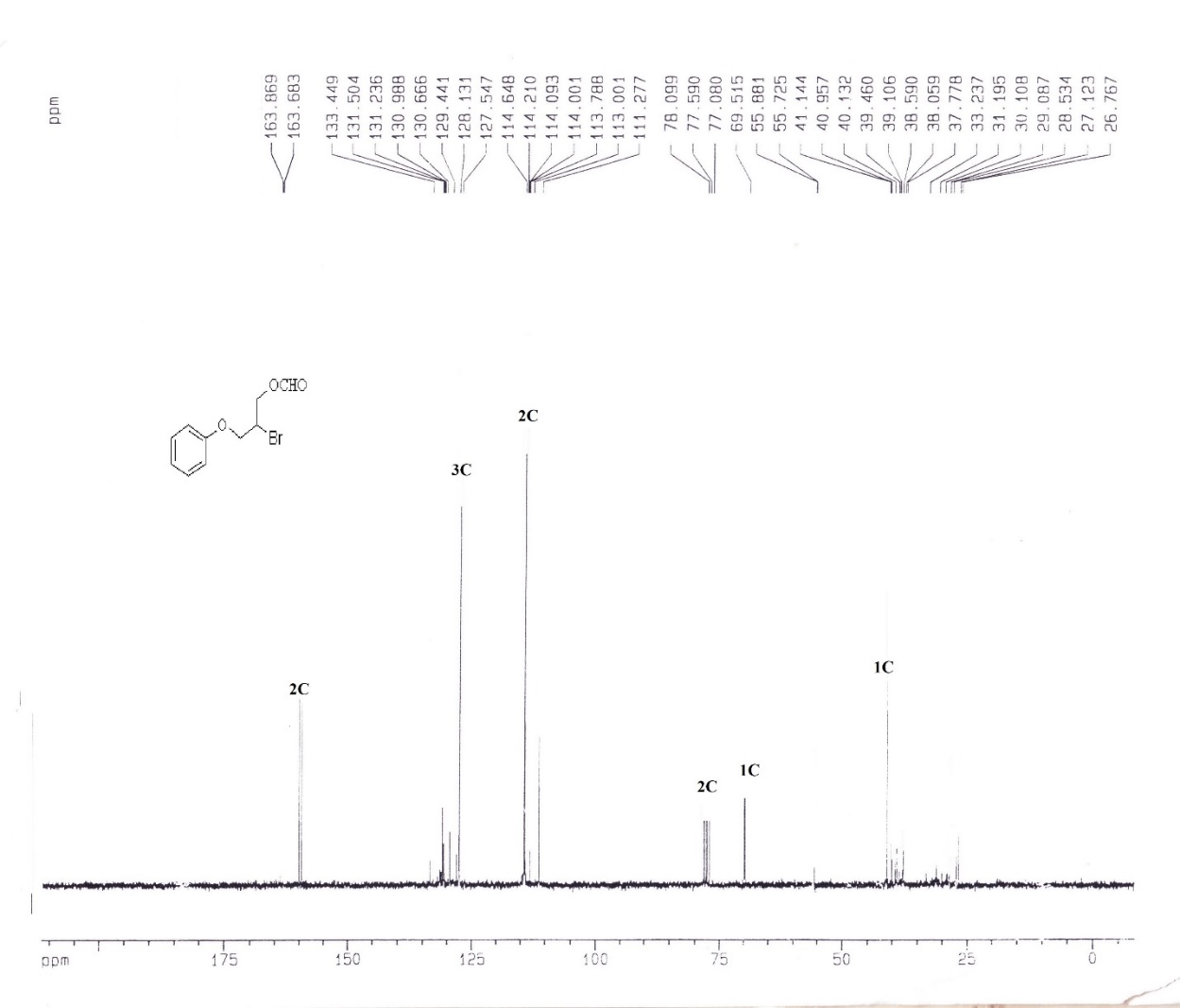


**2.2.3. 1,2-Dibromo-3-isopropoxypropane (2a)**^3^

Light yellow viscous liquid, isolated yield: 96%, CAS number 5423-39-1, b.p: 208-212°C, ^1^HNMR (in CDCl_3,_ 250 MHz): Chemical shifts (δ ppm): 4.18 (m, 1H), 3.9-3.5 (m, 4H), 3.19 (m, 1H), 1.3 (d, J=6.674 Hz, 6H). Anal. Calcd for C_6_H_12_Br_2_O (MW=259.964 g/mol): C, 27.73%; H, 4.66%. Found: C, 27.68%; H, 4.12%. MS(m/z)(g/mol): 259.9291 (100%, C_6_H_12_Br_2_O), 182.9156 (68.7%, C_6_H_12_BrO), 104.0323 (76.9%, C_6_H_12_O), 81.9492 (27.6%, Br), 57.1830 (5.3%, C_3_H_7_).

**2.2.4. 2-Bromo-3-isopropoxypropyl formate (2b)** ^4^

Brown yellow viscous liquid, isolated yield: 89%, b.p: 198-200°C , FTIR (υ/cm^-1^): 4433, 2933, 2892, 2808, 1668, 1350, 1266, 1250, 1000, 800 cm^-1^. ^1^ HNMR (in CDCl_3,_ 250 MHz): Chemical shifts (δ ppm): 7.97 (s, 1H, CHO), 3.75-3.86 (m, 3H), 3.52-3.613.8 (m, 2H), 3.04 (m, 1H), 1.17 (d, J=6.789Hz, 6H). ^13^CNMR Chemical shifts (δ ppm): 161.21 (1C, CHO), 70.45 (1C), 64.26(1C), 45.14(1C), 23.21 (6C). Anal. Calcd for C_7_H_12_BrO_3_ (MW=225.07 g/mol): C, 37.35%; H, 5.82% Found: C, 38.61%; H, 4.98%


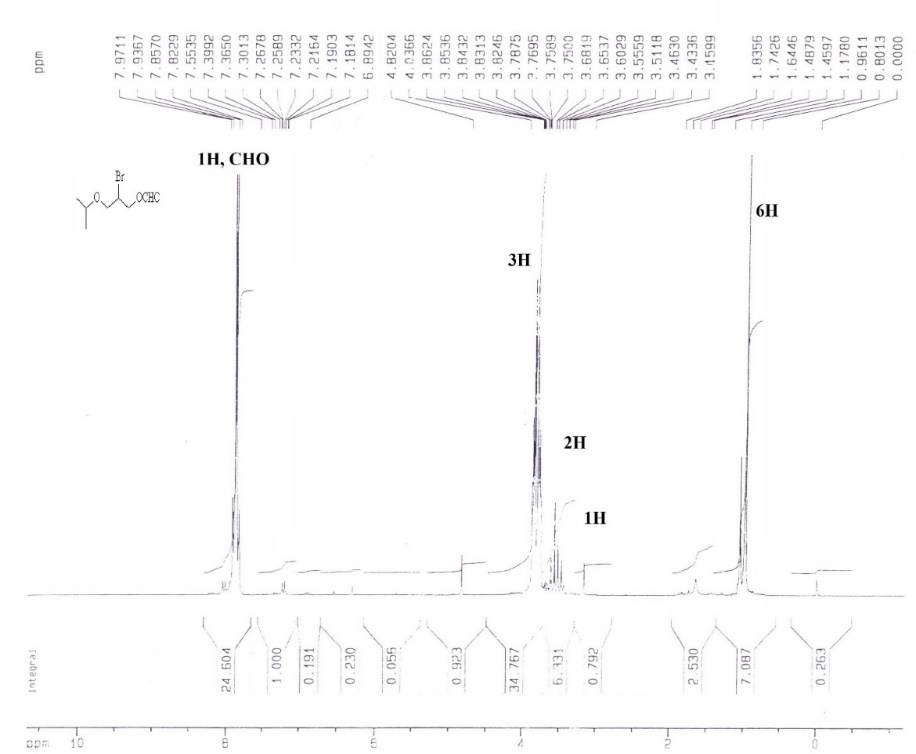

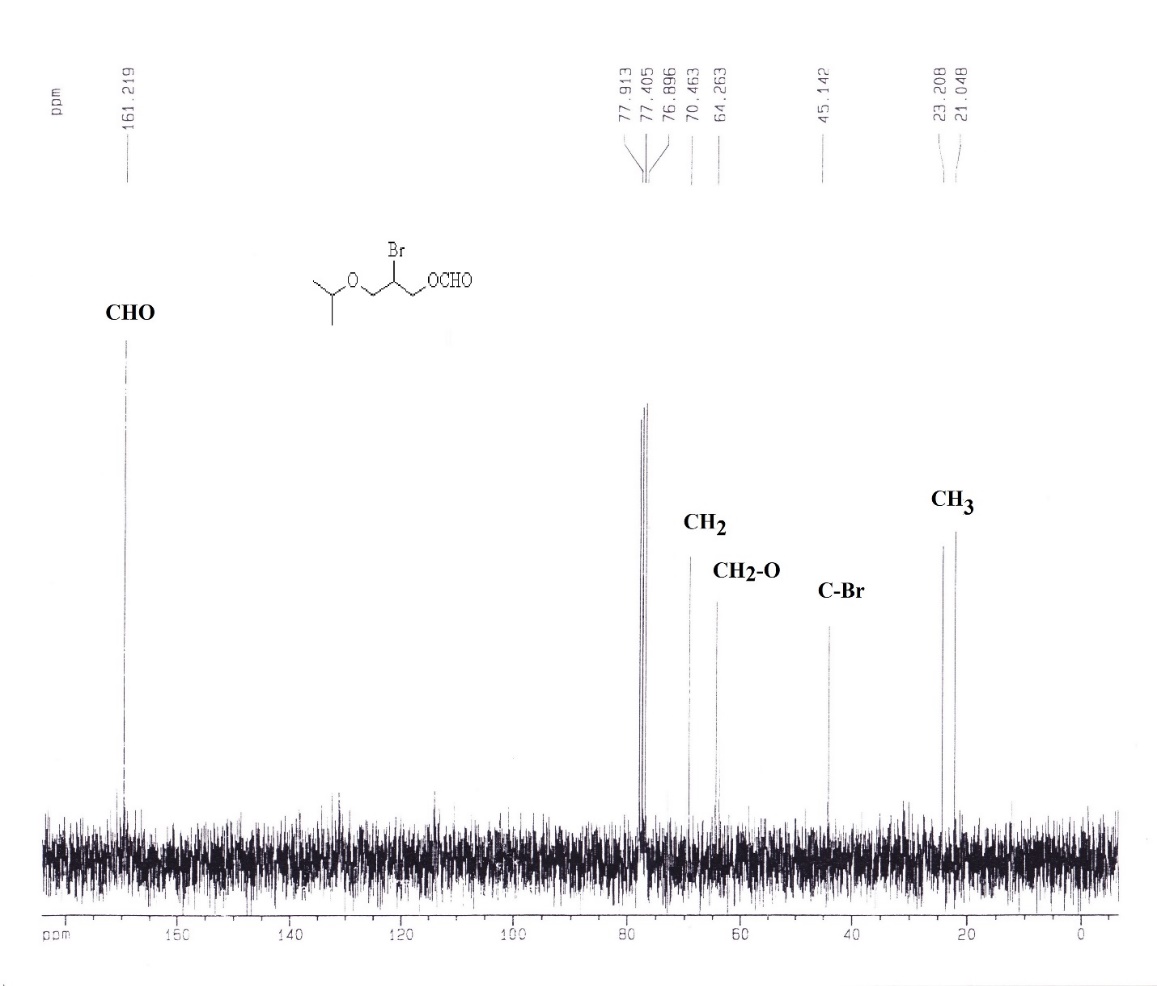


**2.2.5. (1,2-Dibromoethyl) benzene (3a)**^3, 5^

Yellow to brown crystal, isolated yield: 94%, CAS Number: 93-52-7, m.p: 69-72°C, ^1^HNMR (in CDCl_3,_ 250 MHz): Chemical shifts (δ ppm): 7.18-7.33 (m, J =8.525 Hz, J =3.292 Hz, 5H, benzylic), 5.40 (t, J =2.107 Hz, 1H), 4.16-3.93 (d, J =2.147 Hz,2H), ^13^CNMR Chemical shifts (δ ppm): 139.86 (1C),129.91(3C)-129.23(2C), 51.25 (1C) , 39.35(1C). FTIR (υ/cm^-1^) 2968, 2724, 1458, 1303, 1231,1077. Anal. Calcd for C_8_H_8_Br_2_ (MW=263.95 g/mol): C, 36.40%; H, 3.06%; Found: C, 36.41%; H, 3.01%.


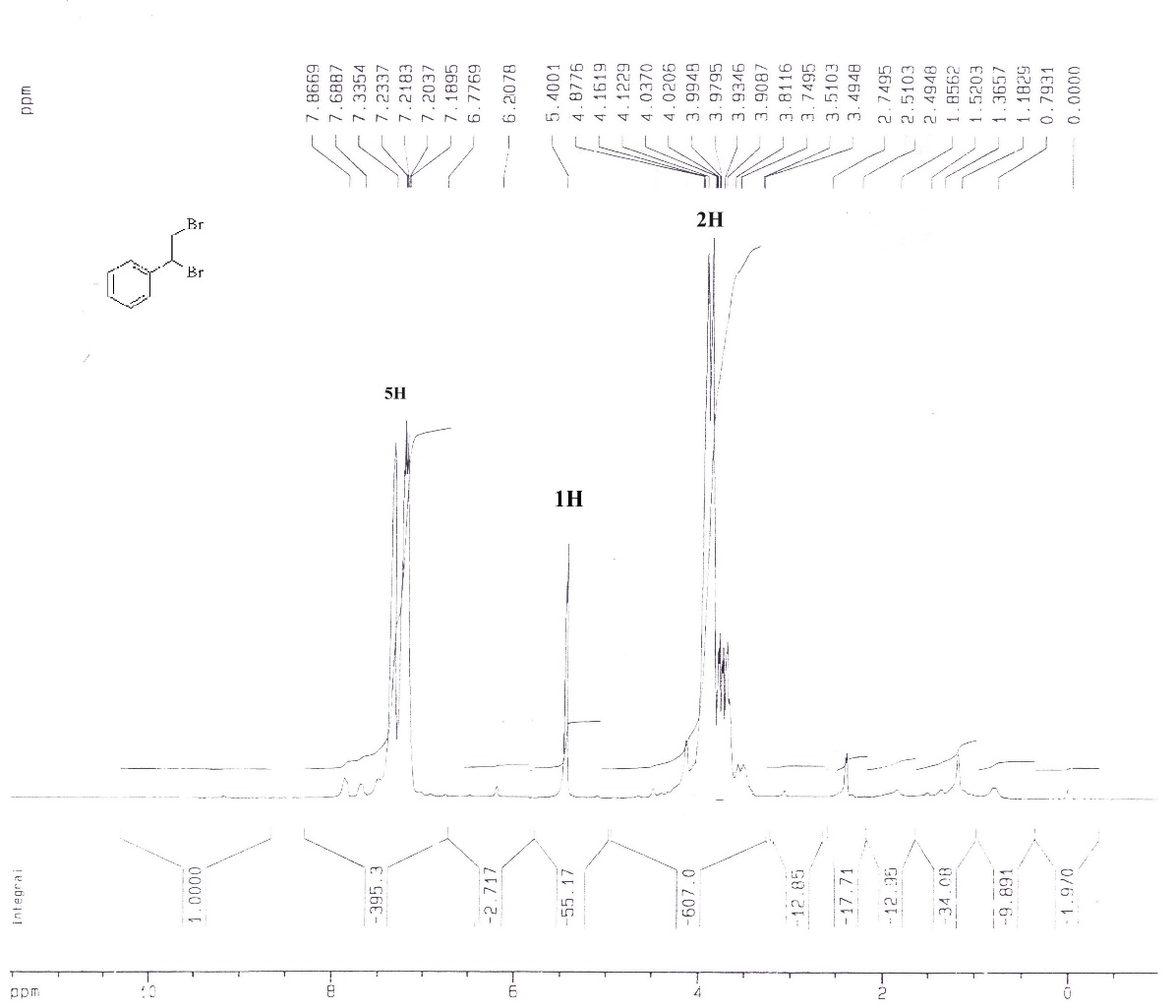


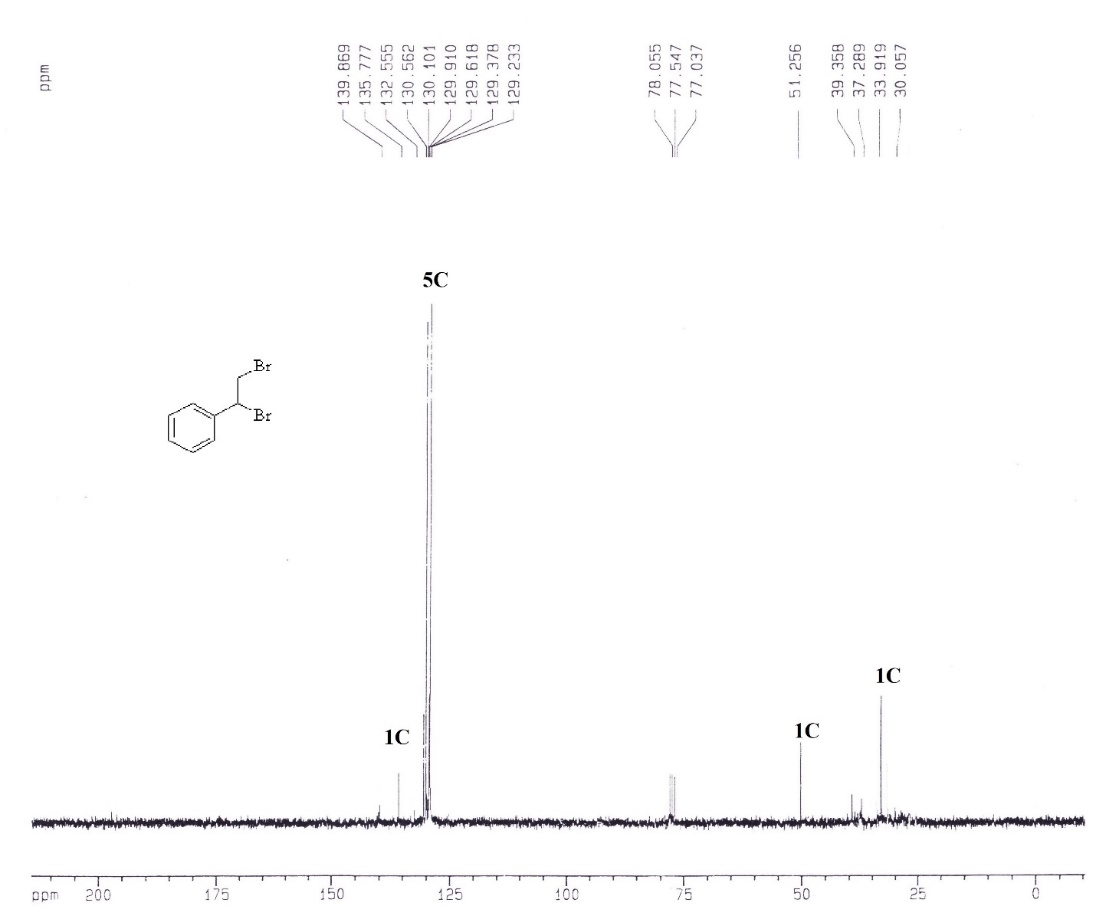


**2.2.6. 2-Bromo-1-phenylethyl formate (3b)** ^4^

Colorless crystal, isolated yield: 90%, m.p: 63-68°C, FTIR (υ/cm^-1^): 3027(=C-H), 2927(C-H), 2873, 1714(C=O), 1452(C=C), 1138(C-O), 756, 703 cm^-1^. ^1^HNMR (in CDCl_3,_ 250 MHz): Chemical shifts (δ ppm): 7.89 (s, 1H, CHO), 7.09-7.34 (5H, J =8.531 Hz, J =3.312 Hz, benzylic), 5.53 (t, J =13.075 Hz, 1H), 3.26-3.18 (d, J =8.225 Hz, 2H) ^13^CNMR Chemical shifts (δ ppm): 162.31 (1C),128.6 (2C),127.95(2C),126.46(1C), 85.55(1C), 39.61(1C). Anal. Calcd for C_9_H_9_BrO_2_ (MW= 229.071 g/mol): C, 47.19%; H, 3.96% . Found: C, 48.12%; H, 3.93%, . MS(m/z)(g/mol): 227.9590 (100%, C_9_H_9_BrO_2_), 210.0419(17.6%, C_9_H_9_Br), 184.0560 (86.5%, C_8_H_7_Br),149.1645 (12.3%), 136.9164 (4.1%, C_8_H_9_O_2_), 94.0011 (16.75%, C_7_H_6_) , 77.1213 (21.3%, C_6_H_5_).


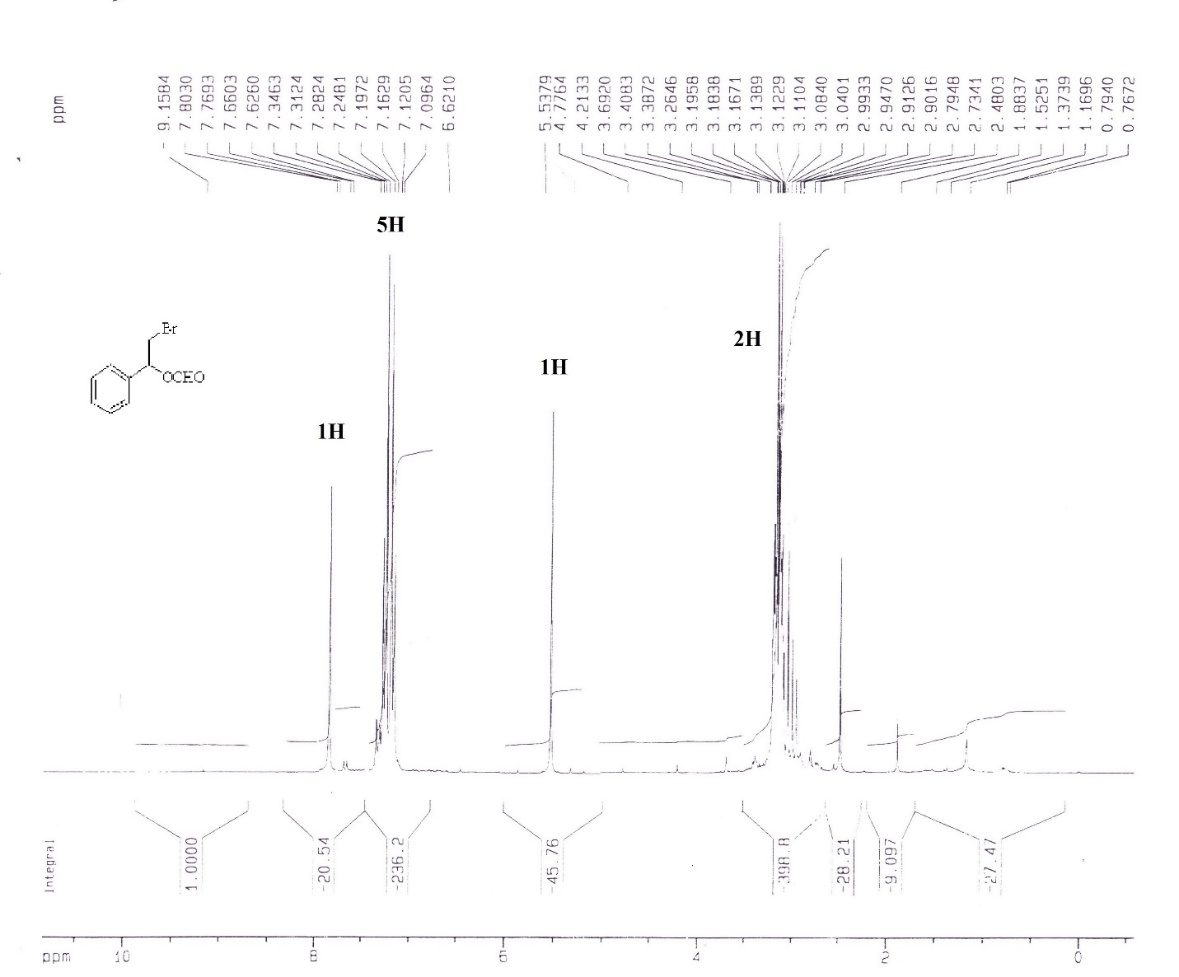


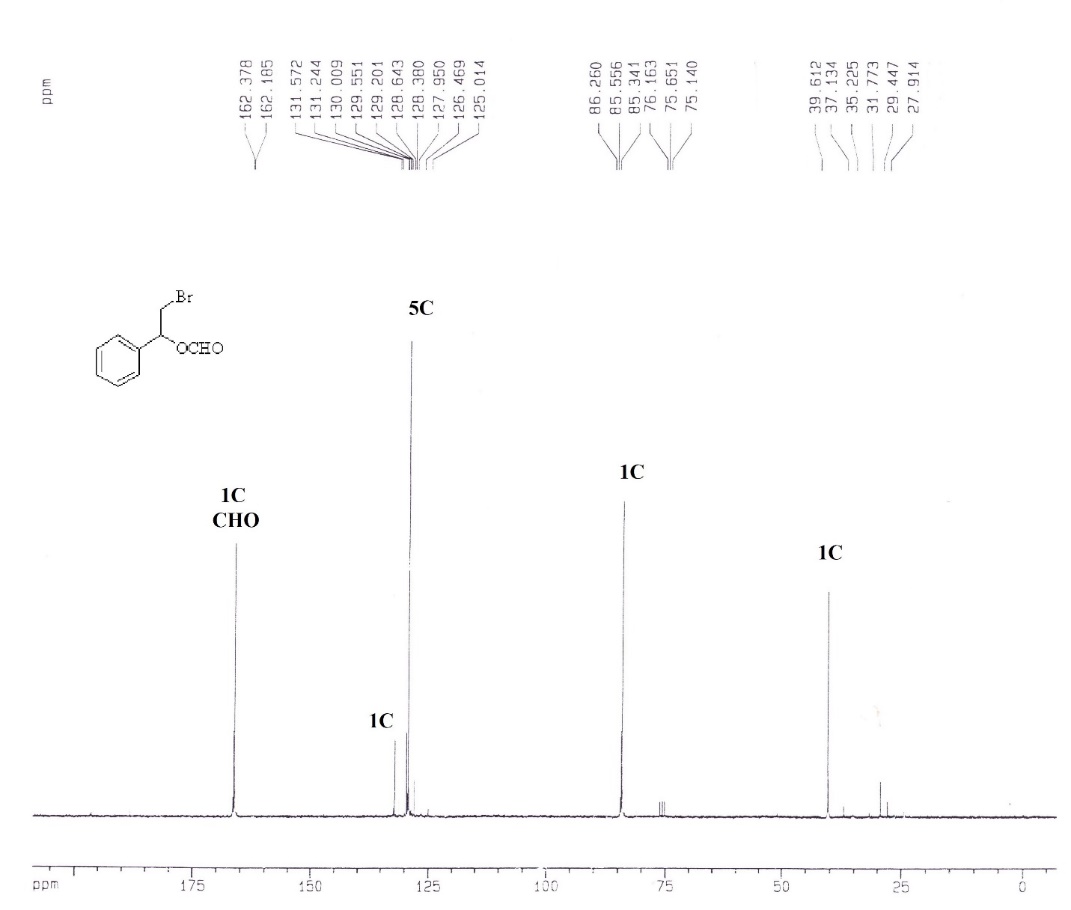


**2.2.7. 2,3-Dibromopropyl methacrylate (4a**)^3, 6, 7^

Brown liquid, isolated yield: 94%, CAS number: 3066-70-4, b.p: 298-300°C, ^1^ HNMR (in CDCl_3,_ 250 MHz): Chemical shifts (δ ppm): ): 6.98-650 (dd, J=7.510 Hz, J=6.975 Hz, 2H), 4.52-4.44 (m, , 3H), 3.65-3.60 (d, J=7.610 Hz, 2H), 2.13-2.10 (s, 3H). ^13^CNMR Chemical shifts (δ ppm): 167.51 (1C, C=O), 137.85 (1C, C=C),127.52 (C=C), 47.58 (1C), 31.67 (1C, C-Br), 20.76 (1C, CH_3_). Anal. Calcd for C_7_H_10_ Br_2_O_2_ (MW= 285.95 g/mol): C, 29.41%; H, 3.52%. Found: C, 28.85%; H, 3.42%.


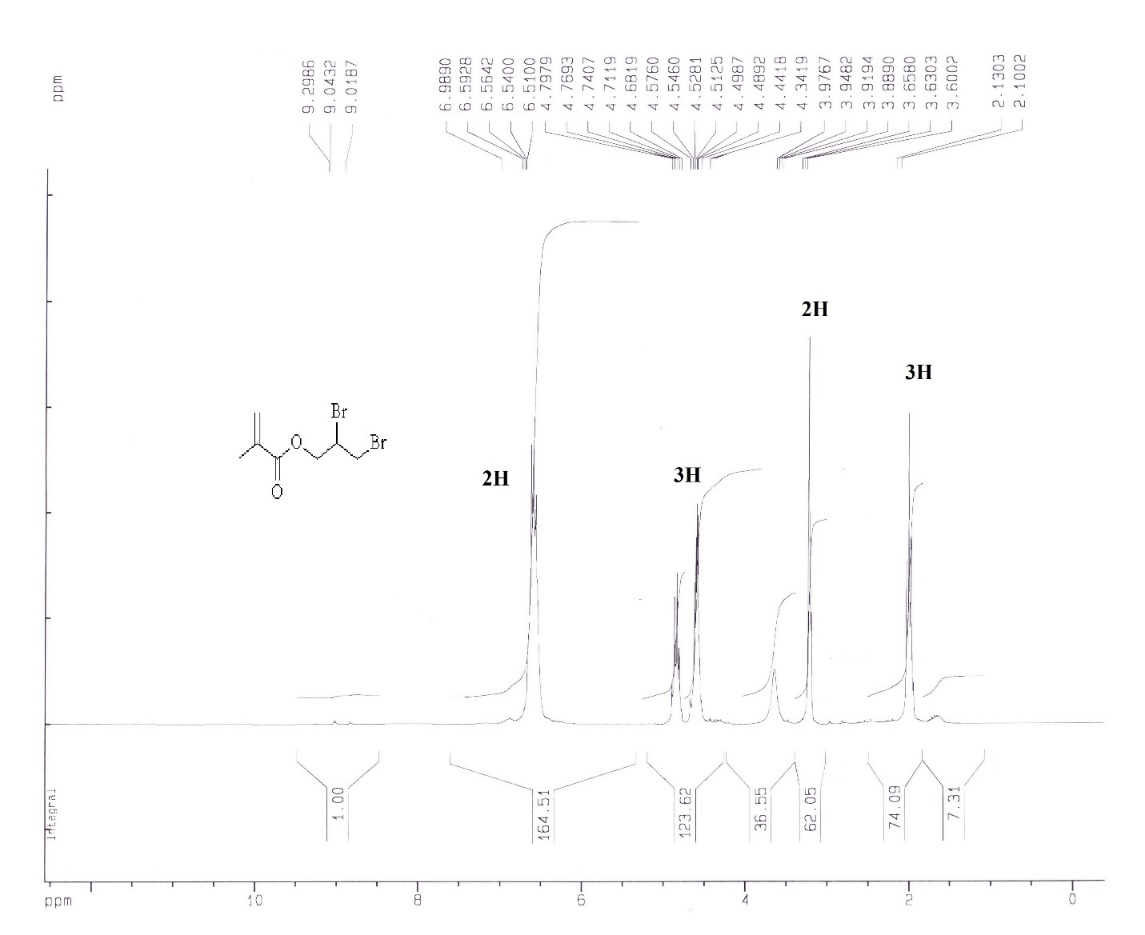


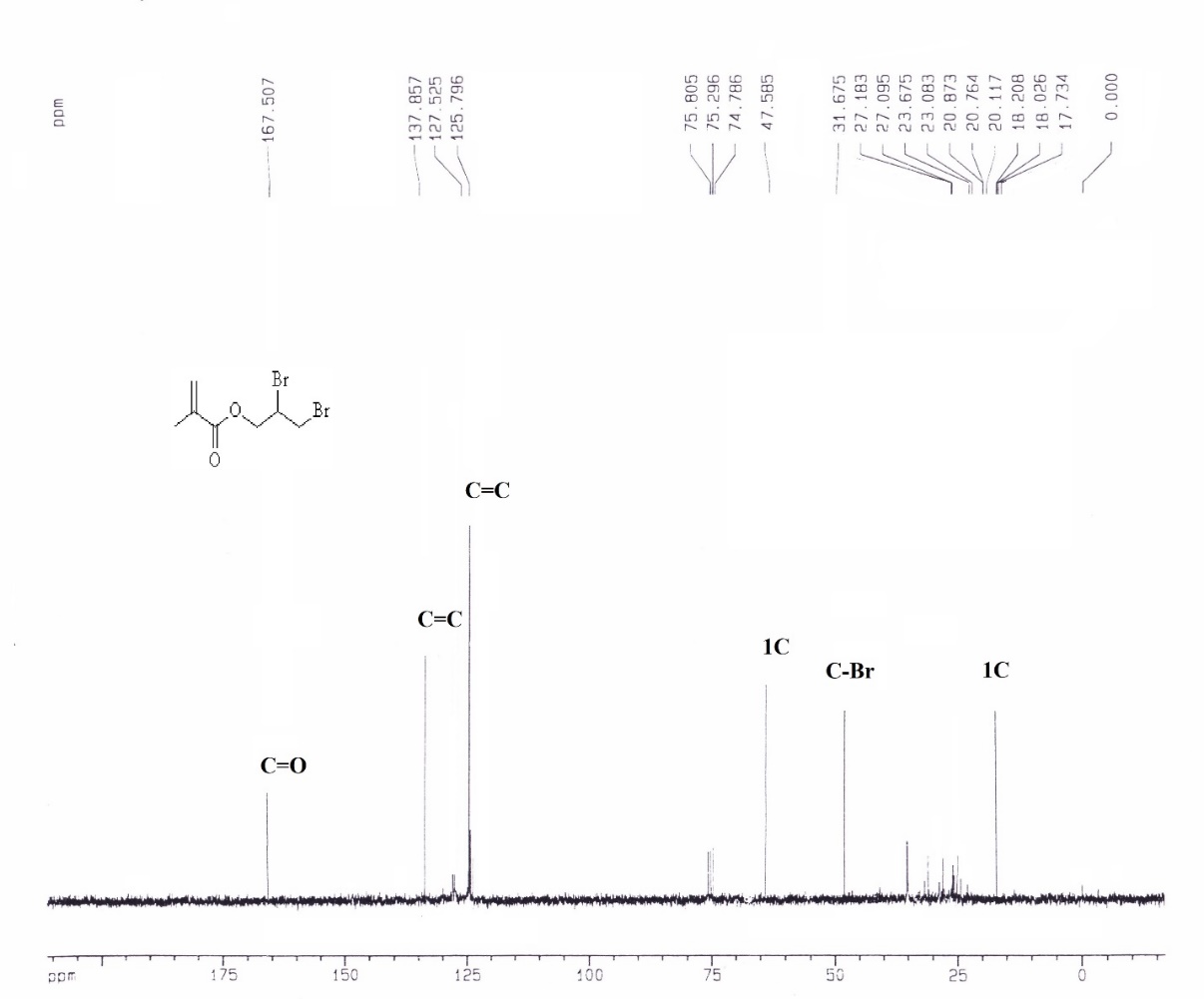


**2.2.8. 2-Bromo-3-(formyloxy)propyl methacrylate(4b)**^4^

Colorless viscous liquid, isolated yield: 92%, b.p: 250-254°C, FTIR (υ/cm^-1^): 3072(=C-H), 2994(C-H), 2857, 1714(C=O), 1642(C=C) ,1421, 1345, 1149(C-O), 927 cm^-1^, ^1^ HNMR (in CDCl_3,_ 250 MHz): Chemical shifts (δ ppm): 7.91 (s, 1H, CHO), 6.81-6.73 (dd, J=8.632 Hz, J=8.622 Hz 2H), 4.72-4.69 (m, 5H), 2.73 (s, 3H). ^13^CNMR Chemical shifts (δ ppm): 166.77 (1C, CHO), 160.92(1C, C=O), 137.97 (1C, C=C),126.77 (C=C), 63.12 (1C, CH_2_), 41.28 (1C, C-Br), 17.89 (1C, CH_3_). Anal. Calcd for C_8_H_11_BrO_3_ (MW= 251.08 g/mol): C, 38.28 %; H, 4.42 %, Found: C, 38.12%; H, 3.96 .


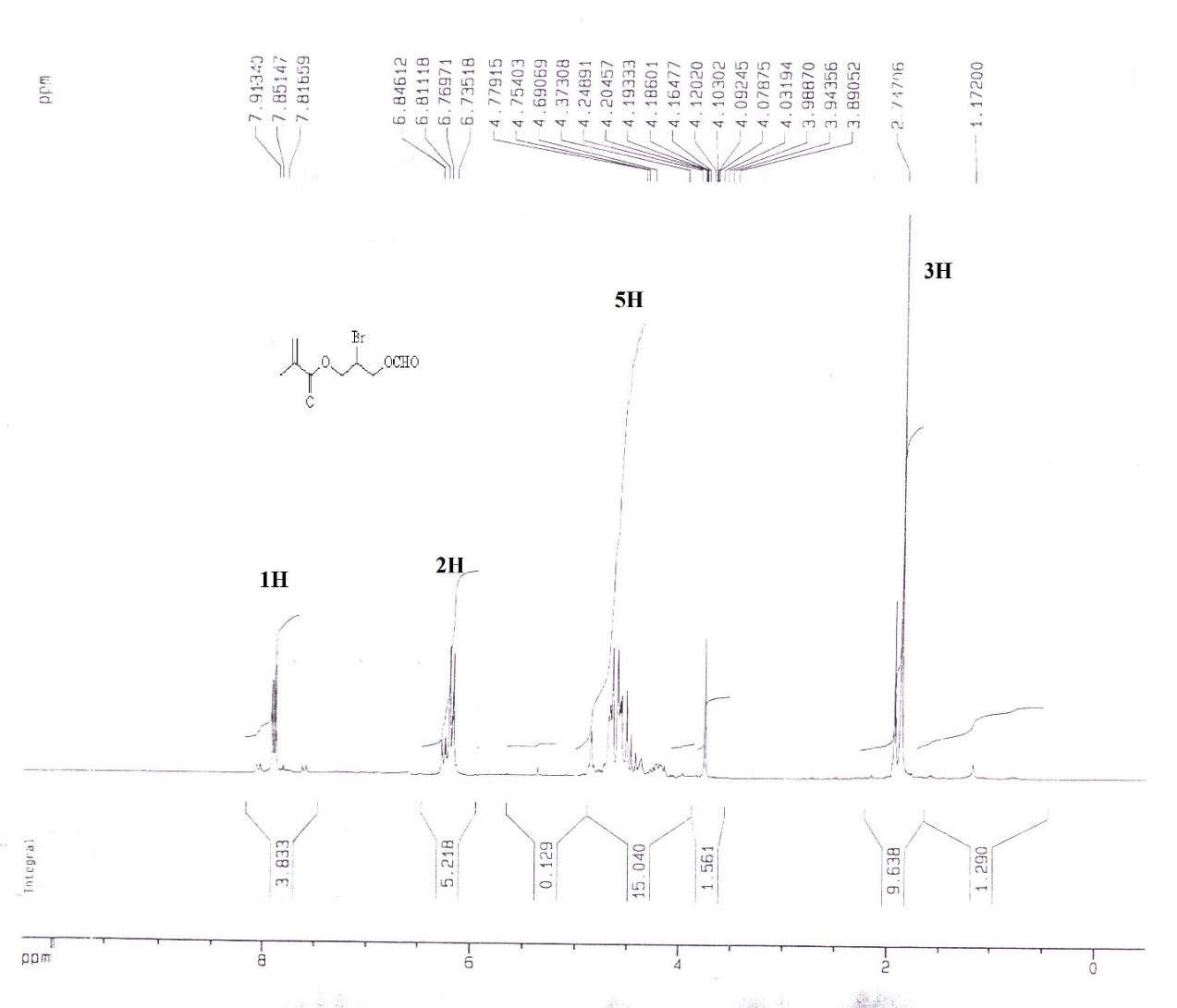


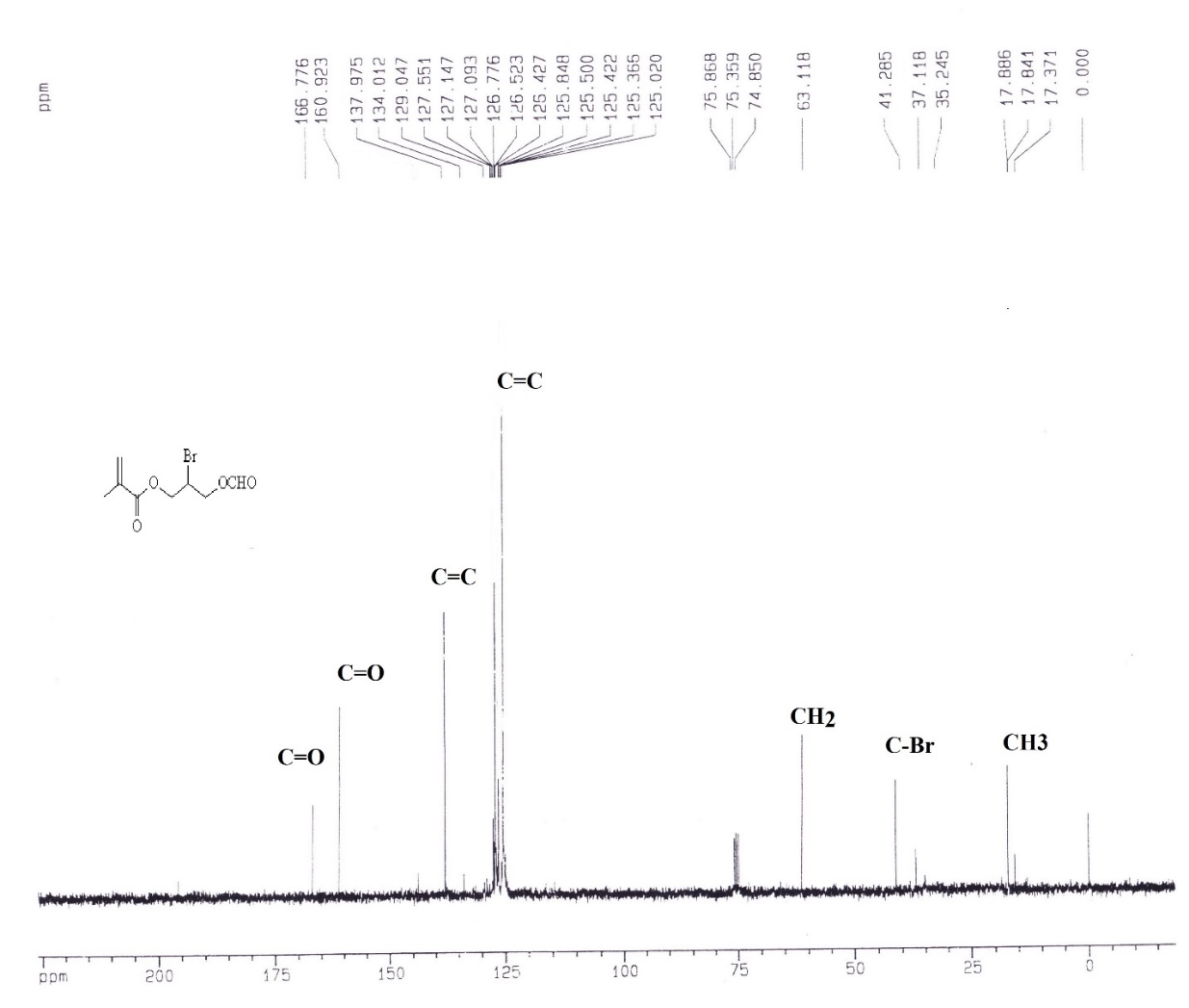


**2.2.9. 2-bromocyclooctyl formate (5b)**^3^

Colorless liquid, isolated yield: 86%, b.p: 183-185°C, ^1^HNMR (in CDCl_3,_ 250 MHz): Chemical shifts (δ ppm): 7.69 (s, 1H, CHO), 4.33-4.20 (q, J=8.765 Hz, 1H), 4.17(q, J=7.751 Hz, 1H), 1.67 (m, 12H). ^13^CNMR Chemical shifts (δ ppm): 160.83 (1C, CHO), 77.41 (1C, CH), 50.72 (1C, C-Br), 36.83 (1C, CH), 29.88-30.52(5C). Anal. Calcd for C_9_H_15_BrO_2_ (MW =234.03 g/mol): C, 45.97%; H, 6.43%. Found: C, 46.37%; H, 6.51%.


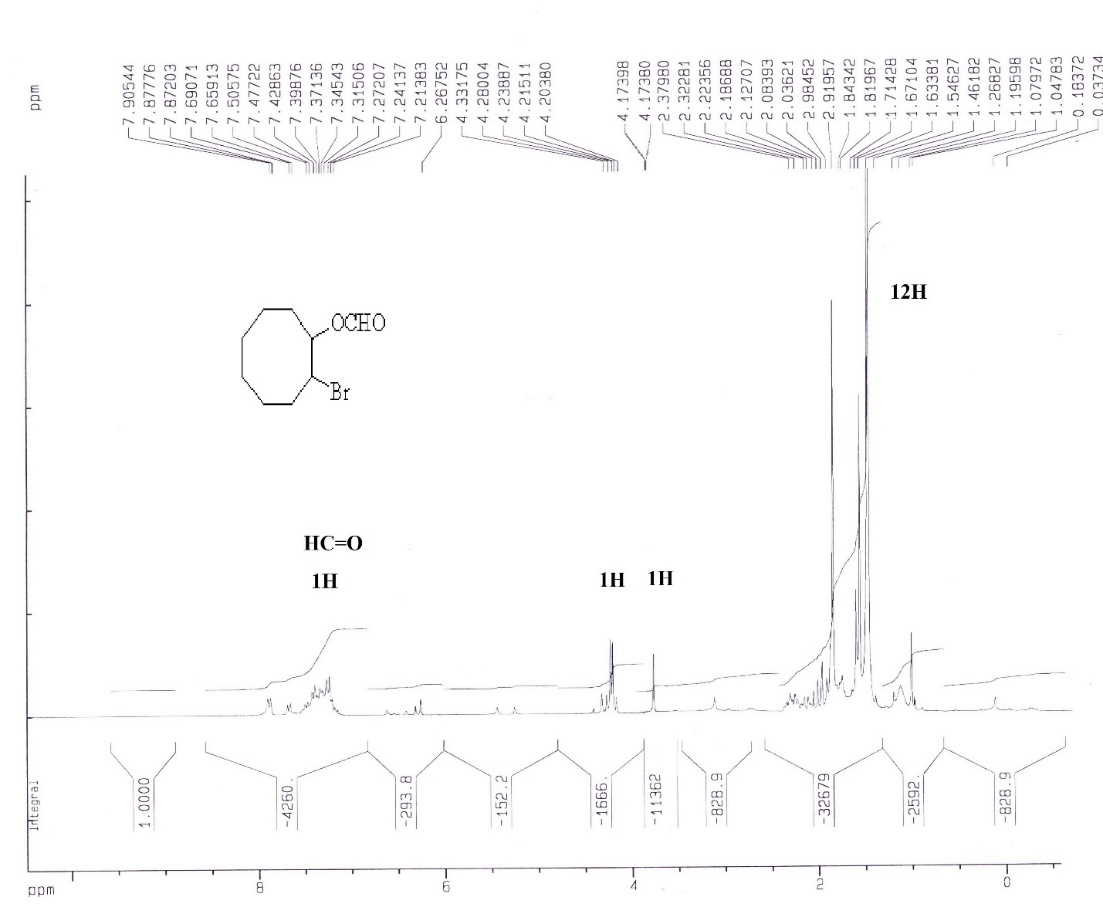


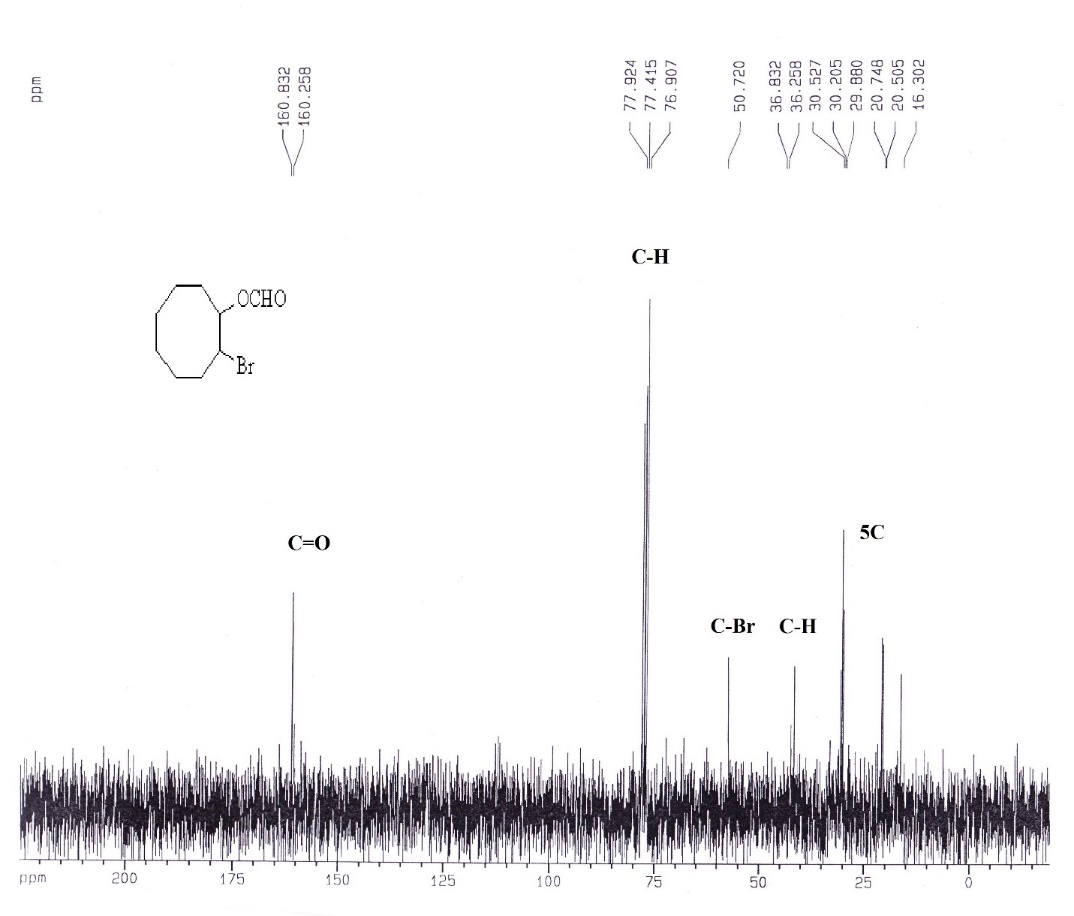


**Figure S1:** Spectra data for products listed in Table 2

## **SI References**

(1) Ebrahimzadeh, F. Synthesis of secondry amines via amination of alcohols with benzylamine using nanocatalyst Fe_3_O_4_@ SiO_2_@CS@ EDTA/Cu(II). *International Research Journal of Modernization in Engineering Technology and Science* **2023**, *5* (10), 2640-2646. DOI: <https://www.doi.org/10.56726/IRJMETS45483>

(2) Ebrahimzadeh, F.; Jamalain, A.; Zaree, S. Core-shell magnetic nanocomposite Fe_3_O_4_@SiO_2_@CS@POCl_2-x_ for alcohols to alkyl halides transformation. *Phosphorus, Sulfur, and Silicon and the Related Elements* **2023**, 1-9. DOI: <https://doi.org/10.1080/10426507.2023.2279614>

(3) Iranpoor, N.; Firouzabadi, H.; Azadi, R.; Ebrahimzadeh, F. Regioselective synthesis of vic-halo alcohols and symmetrical or unsymmetrical vic-dihalides from epoxides using triphenylphosphine N-halo imides. *Canadian journal of chemistry* **2006**, *84* (1), 69-75. DOI: <https://doi.org/10.1139/v05-261>.

(4) Iranpoor, N.; Firouzabadi, H.; Chitsazi, M.; Jafari, A. A. Reactions of epoxides and episulfides with electrophilic halogens. *Tetrahedron* **2002**, *58* (35), 7037-7042. DOI: <https://doi.org/10.1016/S0040-4020(02)00699-3>

(5) Duruskari, G. S.; Asgarova, A.; Aliyeva, K. N.; Musayeva, S.; Maharramov, A. Condensation Products of Aldehydes with Phenylthiazolidine Obtained from (1, 2-Dibromoethyl) benzene. *Russian Journal of Organic Chemistry* **2020**, *56*, 712-715. DOI: <https://doi.org/10.1134/S1070428020040223>.

(6) Zhang, W.; Yang, M. Eine Studie von wasserlöslichen Siloxan-Acryldispersionen für die Verwendung in Aussenanstrichen. *Surface Coatings International Part B: Coatings Transactions* **2005**, *88*, 107-111. DOI: <https://doi.org/10.1007/BF02699541>

(7) Park, H.-S.; Kim, S.-R.; Park, H.-J.; Kwak, Y.-C.; Hahm, H.-S. et al. Preparation and characterization of weather resistant silicone/acrylic resin coatings. *Journal of Coatings Technology* **2003**, *75*, 55-64. DOI: <https://doi.org/10.1007/BF02697923>
